# A Novel Selenocystine-Accumulating Plant in Selenium-Mine Drainage Area in Enshi, China

**DOI:** 10.1371/journal.pone.0065615

**Published:** 2013-06-04

**Authors:** Linxi Yuan, Yuanyuan Zhu, Zhi-Qing Lin, Gary Banuelos, Wei Li, Xuebin Yin

**Affiliations:** 1 Jiangsu Bio-Engineering Research Centre of Selenium, Suzhou, Jiangsu, China; 2 School of Earth and Space Sciences, University of Science and Technology of China, Hefei, Anhui, China; 3 Environmental Sciences Program and Department of Biological Sciences, Southern Illinois University, Edwardsville, Illinois, United States of America; 4 United States Department of Agriculture-ARS, Parlier, California, United States of America; 5 Advanced Lab for Selenium and Human Health, Suzhou Institute for Advanced Study, University of Science and Technology of China, Suzhou, Jiangsu, China; Massey University, New Zealand

## Abstract

Plant samples of *Cardamine hupingshanesis* (Brassicaceae), *Ligulariafischeri (Ledeb.) turcz* (Steraceae) and their underlying top sediments were collected from selenium (Se) mine drainage areas in Enshi, China. Concentrations of total Se were measured using Hydride Generation-Atomic Fluorescence Spectrometry (HG-AFS) and Se speciation were determined using liquid chromatography/UV irradiation-hydride generation-atomic fluorescence spectrometry (LC-UV-HG-AFS). The results showed that *C. hupingshanesis* could accumulate Se to 239±201 mg/kg DW in roots, 316±184 mg/kg DW in stems, and 380±323 mg/kg DW in leaves, which identifies it as Se secondary accumulator. Particularly, it could accumulate Se up to 1965±271 mg/kg DW in leaves, 1787±167 mg/kg DW in stem and 4414±3446 mg/kg DW in roots, living near Se mine tailing. Moreover, over 70% of the total Se accumulated in *C. hupingshanesis* were in the form of selenocystine (SeCys_2_), increasing with increased total Se concentration in plant, in contrast to selenomethionine (SeMet) in non-accumulators (eg. *Arabidopsis*) and secondary accumulators (eg. *Brassica juncea*), and selenomethylcysteine (SeMeCys) in hyperaccumulators (eg. *Stanleya pinnata*). There is no convincing explanation on SeCys_2_ accumulation in *C. hupingshanesis* based on current Se metabolism theory in higher plants, and further study will be needed.

## Introduction

**Figure 1:**
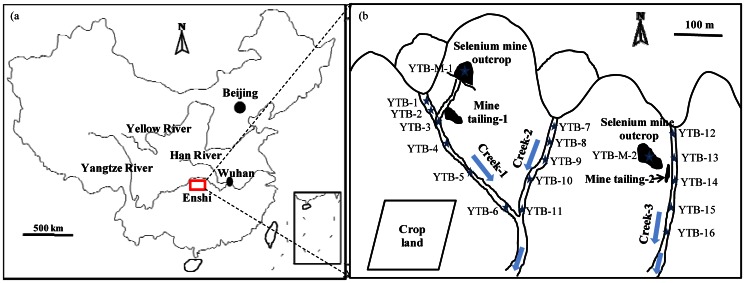
Study site and sampling. A (left): the study area located in Enshi; B (right): 16 sampling sites in selenium mine drainage creeks from Yutangba, Enshi.

Enshi is located in western Hubei province, China (**Fig. 1a**). It has the only selenium (Se) mines in the world, which were formed during the Maokou, Late Permian period. Carbon-siliceous sediment (also called “stone coal”) contains the highest content of Se (up to 8,500 mg/kg), followed by silicon-carbonaceous sediment and then peat coal [1], [2]. Human activities, such as coal mining and agricultural soil amendment with coal ash, have played an important role in the transport and distribution of Se in the local environment [3]. In particular, those processes have substantially increased the bioavailability of Se in soil-plant systems.

Earlier studies indicated that the soil Se concentrations varied significantly in Enshi with the highest Se concentration found in the Se mine drainage areas [4]. High water Se concentrations were also observed at the abandoned “stone coal” spoils and Se mine drainage areas [3]. Previous researches had primarily focused on soil Se bioavailability and Se accumulation in crops in relation to Se toxicity to animals and local residents in Enshi [3], [5]–[10]. However, few studies have been conducted to identify local Se hyper-accumulator species. Since the Se mine drainage areas contain high levels of bioavailable Se, it provides a unique environment to study plants with novel features on Se accumulation and biotransformation. Se hyperaccumulator prince's plume (*Stanleya pinnata*) and twogrooved milkvetch (*Astragalus bisulcatus*) were found to contain Se up to 0.1–1.5% (dry weight) and 0.6% (dry weight), respectively [11]–[14]. The mechanisms responsible for high Se tolerance in *S. pinnata* were found to be related to the levels of ascorbic acid, glutathione, total sulfur, and nonprotein thiols, and may in part be due to increased antioxidants and up-regulated sulfur assimilation [15]. **Pilon-Smits and Quinn (2010)** [16] recently indicated that toxic SeCys can be methylated to form methyl-SeCys, a non-toxic free amino acid, by SeCys methyltransferase (SMT). Because methyl-SeCys does not enter proteins, it can be safely accumulated to high levels in plant tissues, which explains in part the high tolerance of hyperaccumulators to Se.

The specific objectives of this study were to identify local Se-accumulating plant species, and to determine the dominant chemical forms of Se accumulated in plant tissues of different species in Enshi, China. The research findings of this study could be helpful in the development and application of Se phytoremediation and biofortification technologies.

## Study Site and Methods

### Ethics statement

Permissions for field work were obtained from local government. As this was a purely scientific study, no specific permit was needed. This field study did not involve endangering to protected species.

### Study site and sampling

The study site was located in Yutangba (E 109°48′31″, N 30°09′27″, H 1758 m), northwestern Enshi, Hubei Province, including three Se mine drainage creeks (**Fig. 1b**). During the dry season of March to June, the creeks are recharged entirely from groundwater permeating the Se mine as Se drainage water. Zhu et al. (2008) [3] reported that Se mine mainly distributed between the overlying and the underlying layers of muddy shale, mudstone and limestone. The layers were natural barriers that limited the transport of Se drainage in other directions. The creeks formed by seeps were the main natural pathway for Se to transport from the Se mine into the drainage basin. There are three creeks that were sampled (i.e., Creek 1, Creek 2 and Creek 3) (**Fig. 1b**).

There are two Se mine outcrops in Yutangba, Enshi (**Fig. 1b**). One (YTB-M-1) was suspended in 2006 after 5 years in operation, and the mine tailings were discarded 100 meters away. The other (YTB-M-2) was started in 2008 and was still in operation when the samples were collected. There were two common plant species which were collected from the creeks and the banks, including *Cardamine hupingshanesis* (Brassicaceae) and *Ligulariafischeri (Ledeb.) turcz* (Steraceae). These two plant species were collected along with their underlying top sediment samples (0–3 cm depth). The collected samples and their locations are shown in **Table 1** **and** **Figure 1b**.

**Table 1:** The sampling sites and collected plant and sediment samples in Yutangba, Enshi in May 2011.

Sampling Site*	Plant species and code	Sediment code
YTB-1	*Cardamine hupingshanesis* (Brassicaceae)	S1
YTB-2	*Cardamine hupingshanesis* (Brassicaceae)	S1
YTB-3	*Cardamine hupingshanesis* (Brassicaceae)	S1
YTB-4	*Cardamine hupingshanesis* (Brassicaceae), *Ligulariafischeri (Ledeb.) turcz* (Steraceae)	S1, S2
YTB-5	*Cardamine hupingshanesis* (Brassicaceae), *Ligulariafischeri (Ledeb.) turcz* (Steraceae)	S1, S2
YTB-6	*Cardamine hupingshanesis* (Brassicaceae), *Ligulariafischeri (Ledeb.) turcz* (Steraceae)	S1, S2
YTB-7	*Cardamine hupingshanesis* (Brassicaceae)	S1
YTB-8	*Cardamine hupingshanesis* (Brassicaceae), *Ligulariafischeri (Ledeb.) turcz* (Steraceae)	S1, S2
YTB-9	*Cardamine hupingshanesis* (Brassicaceae), *Ligulariafischeri (Ledeb.) turcz* (Steraceae)	S1, S2
YTB-10	*Cardamine hupingshanesis* (Brassicaceae), *Ligulariafischeri (Ledeb.) turcz* (Steraceae)	S1, S2
YTB-11	*Cardamine hupingshanesis* (Brassicaceae)	S1
YTB-12	*Cardamine hupingshanesis* (Brassicaceae)	S1
YTB-13	*Cardamine hupingshanesis* (Brassicaceae)	S1
YTB-14	*Cardamine hupingshanesis* (Brassicaceae)	S1
YTB-15	*Cardamine hupingshanesis* (Brassicaceae)	S1
YTB-16	*Cardamine hupingshanesis* (Brassicaceae)	S1

Note:

* The sampling locations were shown in Figure 1b.

### Sample analysis

#### Sample preparation

Plant samples were washed in tap water to remove soils from the root surface, and then rinsed in deionized water. Plants were separated into roots, stem, and leaves, oven-dried at approximately 50°C for 24 hours, and ground to pass through a 0.2 mm sieve (FT-100, China). Sediment samples, free of plant roots and detritus, were oven-dried at approximately 50°C for 24 hours, and then ground in an agate-mortar to pass through a 0.15 mm sieve.

#### Measurement of total Se

0.5–3.0 g samples were weighed into a 50 ml conical flask. Ten ml of concentrated HNO_3_ and HClO_4_ (4∶1, v/v) were added to each flask and covered with a glass funnel. The flasks were kept overnight at the room temperature, heated at 100°C for one hour, 120°C for two hours, and 180°C for one hour on an electrical hot plate. The samples were then heated at 210°C until the white fume formed and the volume of solution was approximately 2 ml. After acid digestion, the digest was cooled to room temperature and 5 ml HCl (12 M) was added to reduce Se(VI) to Se(IV) for 3–4 h as following pathway: H_2_SeO_4_+2HCl (Concentrated) = H_2_SeO_3_+Cl_2_+H_2_O [17]. Then, the digestion solution was brought up to 25 ml for Se analysis. The detailed procedure was described by **Gao et al. (2011**) [18]. The total Se concentration was determined by Hydride Generation Atomic Fluorescence Spectrometry (HG-AFS 9230) (Beijing Titan Instrument Co., China). National standard reference materials GSV-1 (shrub leaves) and GSS-1 (soil) were used for plant samples and soil samples, respectively. The recovery of the standard reference materials ranged from 85.5% to 117.8%, and the relative standard deviation (RSD) of reference materials was calculated as 0.76%. The instrument detection limit (DL) was 0.08 µg/kg.

#### Measurement of Se speciation

The leaf, stem and root samples were extracted with 100 mM Tris-HCl buffer (pH 7.5) in an ultrasonic tank for 10 min. The enzyme Protease XIV was added and the mixture was shaken for 24 h at 37°C. After extraction, the mixture was centrifuged at 10000 rpm/min for 30 min at 4°C. The supernatant was collected and filtered through a 0.22 µm filter for Se speciation analysis. The separation of different Se compounds was performed using a Hamilton PRP X-100 anion exchange column (4.1 mm×250 mm×10 µm). The mobile phase was 40 mM NH_4_H_2_PO_4_ (pH 6.0) with a flow rate of 1 mL/min. The eluent from the column was mixed with concentrated HCl (flow rate: 3 mL/min) and then passed through the UV unit. 1.2% NaBH_4_ in 0.1 mol/L NaOH (flow rate: 3 mL/min) was added after the UV unit. Argon was used as carrier gas (260 mL/min) to transfer H_2_Se from the gas liquid separator through the dryer into the AFS detector (SAP-10-AFS-9230, Titan Co, Beijing). The dryer gas was nitrogen with a flow rate of 3 L/min. The organic Se standards (L-Selenocystine, Se-Methylseleno-L-cysteine, DL-Selenomethionine) were purchased from Tokyo Chemical Industry, Co., Japan, and the inorganic Se standards (Selenite and Selenate) were purchased from National Reference Material Centre, China. Since Se(VI) could be reduced to Se(IV) by concentrated HCl during elution, the inorganic Se of sample was represented by measured Se(IV). Thus, four Se species were detected in plant tissues, including selenocystine (SeCys_2_), selenomethylselenocysteine (SeMeCys), selenomethionine (SeMet) and Se(IV) species were determined with precisions of 5%, 6%, 10% and 5%, respectively. The instrument detection limits were 2 µg/L, 5 µg/L, 10 µg/L and 2 µg/L (100 µL injection, 10 times of the baseline noise), respectively. The detailed description of the procedure was given by **Liang et al. (2006)** [19] **and Mazej et al. (2006)** [20]. It should be pointed out that there is lack of Selenocysteine (SeCys) standard because of its high instability due to oxidization in air [21], and the alternative reference material, Selenocystine (SeCys_2_), was used since the SeCys could be co-eluted with SeCys_2_ in LC-UV-HG-AFS [22]. Thus, the determined concentrations of SeCys_2_ here will be the total concentrations of SeCys and SeCys_2_ in plant tissues.

### Statistical Analysis

Normality test, two sample t test on normally distributed data, nonparametric test (Two sample Kolmogorov-Smirnov Test/K-S Test) on non-normally distributed data, and correlation analyses were performed by OriginPro 8.

## Results

### Total Se in plant tissues

**Figure 2:**
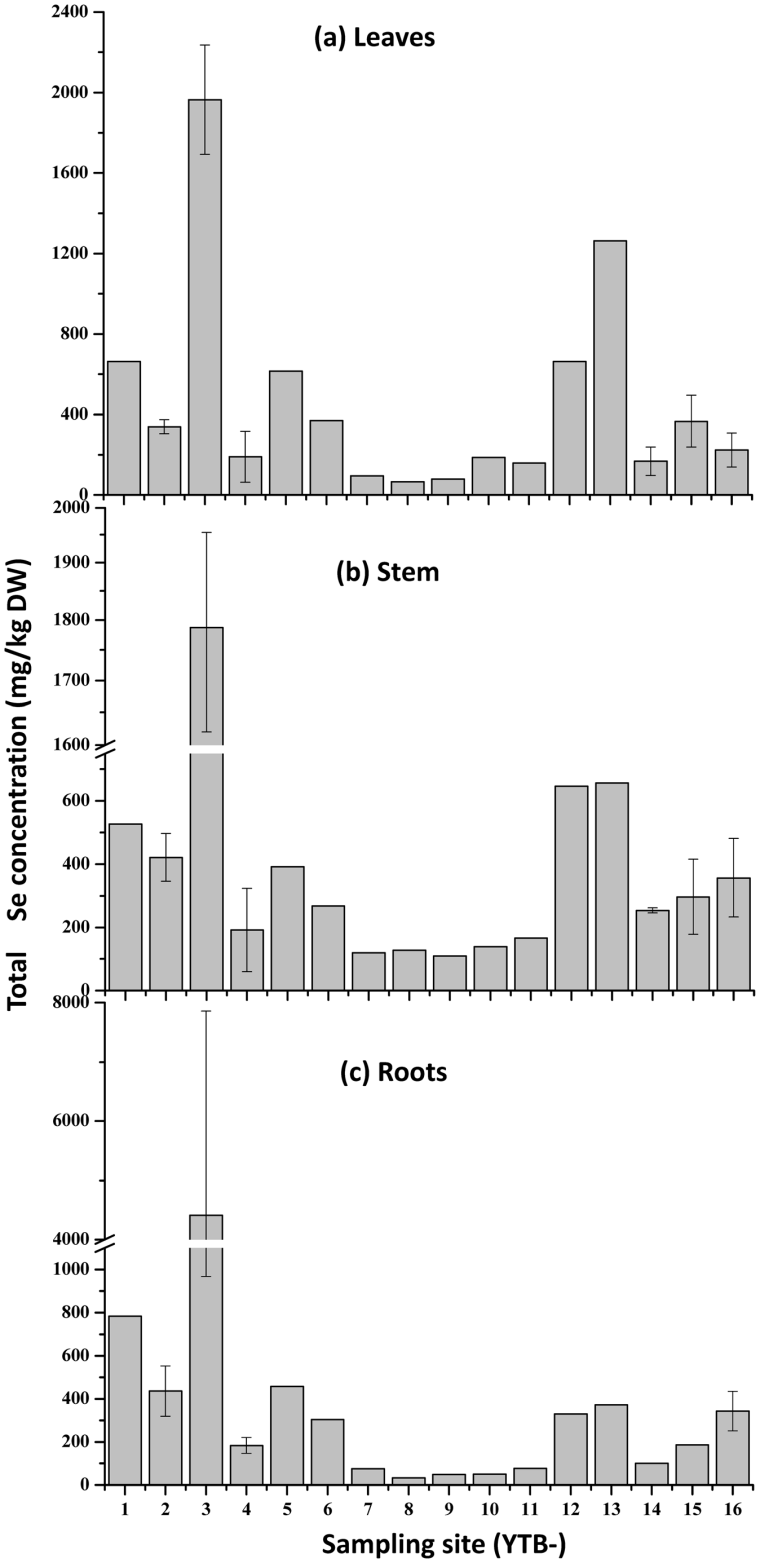
Concentrations of total Se in different tissues of *Cardamine hupingshanesis* (Brassicaceae). Top: leaves; Middle: stem; Bottom: roots. The error bar was calculated on triplicate samples.

Concentrations of total Se accumulated in plant tissues of *C. hupingshanesis* are shown in **Fig. 2**. The highest plant Se accumulation was observed at the sampling site 3 (or YTB-3) in Creek 1 (YTB 1–6 sampling sites, see **Fig. 1**), showing 1965±271 (n = 3) mg/kg DW in leaves, 1787±167 (n = 3) mg/kg DW in stem, and 4414±3446 (n = 3) mg/kg DW in roots. Relative lower Se concentrations in *C. hupingshanesis* were found in Creek 2 (YTB 7–11 sampling sites), with 56.85±19.20 mg/kg DW (n = 5) in roots, 132.67±21.48 mg/kg DW (n = 5) in stems, and 117.70±52.84 mg/kg DW (n = 5) in leaves. Concentrations of total Se in *L. (Ledeb.) turcz* (Steraceae) at YTB 4–6 and 8–10 sampling sites were compiled in **Table 2**. Concentrations of total Se in *L. (Ledeb.) turcz* grown in Creeks 1 and 2 (n = 6) were 18.98±8.13 mg/kg in root, 14.01±7.78 mg/kg in stem, and 25.62±18.05 mg/kg in leaves.

**Table 2:** Concentrations (unit: mg/kg DW)of total selenium (TSe) in sediment and plant tissues of *Ligulariafischeri (Ledeb.) turcz* (Steraceae), along with selenium speciation in plant tissues, at different sampling sites in Yutangba, Enshi, China.

Site*	Sediment Se concentration	Plant tissue	Plant Se concentration				
			TSe	SeCys_2_	SeMeCys	SeMet	Se(IV)
YTB-4	42.51	leaf	31.25	<DL	2.77	28.23	<DL
		stem	17.52	<DL	2.74	14.26	<DL
		root	21.75	<DL	4.62	16.38	<DL
YTB-5	15.13	leaf	51.55	/	/	/	/
		stem	26.66	/	/	/	/
		root	29.51	/	/	/	/
YTB-6	8.21	leaf	39.12	3.59	10.68	20.85	3.88
		stem	13.06	0.64	3.47	8.72	0.18
		root	12.83	<DL	1.90	10.43	0.67
YTB-8	29.39	leaf	9.31	<DL	3.51	5.79	<DL
		stem	9.72	<DL	1.69	5.71	2.33
		root	16.77	0.89	7.13	5.15	3.60
YTB-9	45.56	leaf	5.64	<DL	1.54	1.23	2.87
		stem	3.48	<DL	0.89	2.53	<DL
		root	7.64	<DL	1.68	3.36	2.60
YTB-10	31.90	leaf	16.87	1.45	6.05	5.60	3.77
		stem	13.59	<DL	3.95	9.64	<DL
		root	25.37	0.58	6.91	13.34	4.54

Samples were collected in May 2011.

Note: “*: The sampling locations were shown in Figure 1b. “/” No sample to be determined. “<DL” Below detection limit value.

The statistical analysis revealed that *C. hupingshanesis* has significant higher Se concentrations in leaves (432±471) (n = 27) (mg/kg DW), stems (425±447) (n = 27) (mg/kg DW), and roots (462±709) (n = 19) (mg/kg DW) than those of *L. (Ledeb.) turcz* in leaves (25.62±18.05) (n = 6) (mg/kg DW) (K-S Test, D = 1, Z = 0.45, P<0.01), stems (14.01±7.78) (n = 6) (mg/kg DW) (K-S Test, D = 1, Z = 0.45, P<0.01), and roots (18.98±8.13) (n = 6) (mg/kg DW) (K-S Test, D = 1, Z = 0.46, P<0.01), respectively. In contrast, there were no significant differences (K-S Test, P>0.1) in Se distributions in plant tissues of *C. hupingshanesis*.

### Total Se in sediments

**Figure 3:**
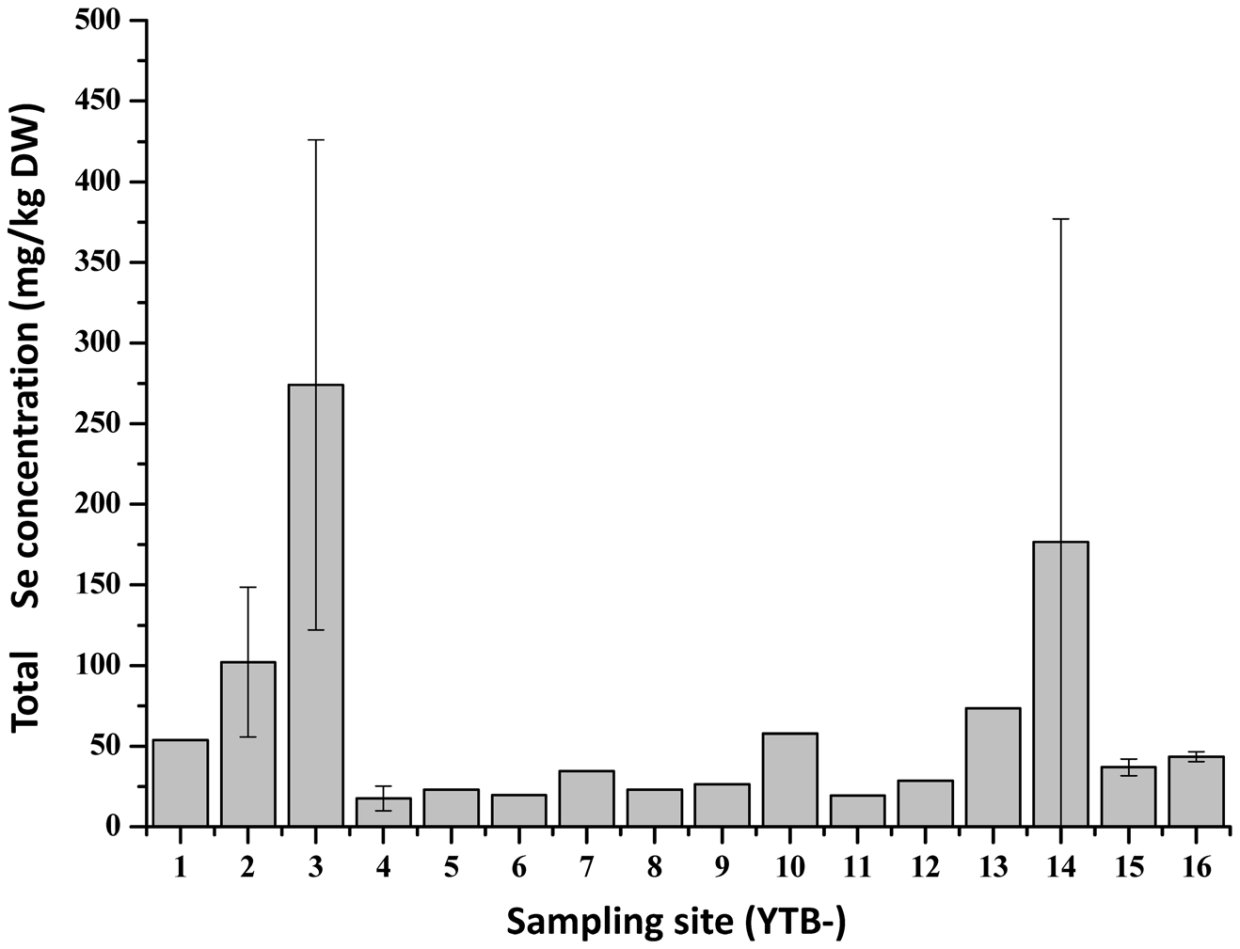
Total Se content in underlying top sediment of *Cardamine hupingshanesis* (Brassicaceae). The error bar was calculated on triplicate samples.

Total Se concentrations in underlying top sediments (0–3 cm depth) of *C. hupingshanesis* and *L. (Ledeb.) turcz* are shown in **Figure 3** and **Table 2**, respectively. The sediments Se concentrations generally varied from 10 to 70 mg/kg DW, except for those sampling sites near Se mine tailings (YTB 3 and 14) where sediment Se concentrations were 274±152 (n = 3) and 177±200 (n = 3) mg/kg DW, respectively. Since these two plants were only co-existed at six sampling sites (YTB 7–9, YTB 11–13), the calculated average sediment Se concentrations on those points were 25.39±14.16 (n = 8) mg/kg DW for C. *hupingshanesis* and 28.78±14.76 (n = 6) mg/kg DW for *L. (Ledeb.) turcz*, and there was no significant (K-S Test, D = 0.54, Z = 0.29, P = 0.19) difference between them though the total Se concentrations in C. *hupingshanesis* tissues were significant higher than those of *L. (Ledeb.) turcz* tissues.

### Selenium speciation in plant tissues

**Figure 4:**
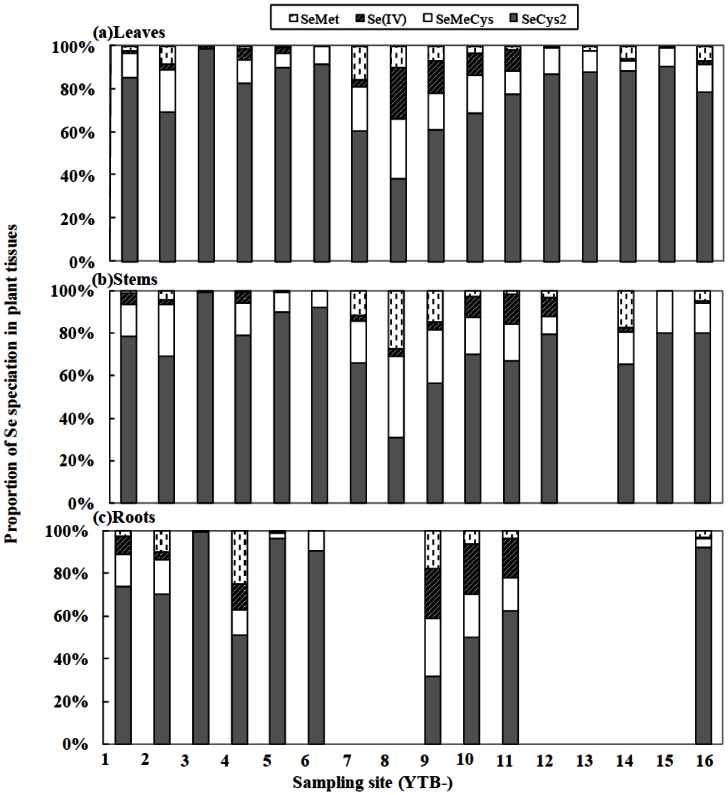
Selenium speciation in different tissues of *Cardamine hupingshanesis* (Brassicaceae). Top: leaves (n = 16); Middle: stem (n = 15); Bottom: roots (n = 10). The speciation analysis was only conducted when the plant had enough tissues samples of root, stem and leaf.

The chemical compositions of Se in *C. hupingshanesis* are shown in **Figure 4**, while the Se speciation in *L. (Ledeb.) turcz* is presented in **Table 2**. The dominant chemical compounds of Se in *C. hupingshanesis* included SeCys_2_ with 78±16% in leaves, 74±17% in stems, and 78±23% in roots, followed by SeMeCys with 12.91±6.74% in leaves, 16.65±9.21% in stems, and 12.43±8.88% in roots, and Se(IV) with 5.12±6.77% in leaves, 4.07±4.31% in stems, and 8.71±10.23% in roots. Moreover, the accumulation of SeCys_2_ was greater in the plant having higher contents of total Se. The Se speciation in *C. hupingshanesis* also varied among different sampling sites. At Site 3 (or YTB 3) where the highest plant Se concentrations were observed, SeCys_2_ accounted for almost all the Se accumulated in *C. hupingshanesis*. In contrast, the lowest proportion of SeCys_2_ in *C. hupingshanesis* was recorded at YTB 8 sampling site, with 30.37% in stem and 38.37% in leaves, which were corresponding to the lowest total Se contents with 128 mg/kg DW in stem and 66.67 mg/kg DW in leaves. Furthermore, there were no significant differences in Se speciation of plant tissues between roots and leaves (Two Sample t Test, t = 0.50, Df = 22, P>0.05), and between stem and leaves (Two Sample t Test, t = 0.64, Df = 27, P>0.05).

The Se speciation results in *L. turcz* were compiled in **Table 2**. SeMet and SeMeCys compounds accounted for 52.34±26.94% and 27.44±21.27% (n = 3) of the total Se accumulated in leaves, 70.90±9.25% and 23.06±10.57% in stems, and 57.10±21.56% and 25.67±13.53% in roots, respectively. Overall, SeMet was the dominant Se compound in *L. turcz*, followed by SeMeCys (**Table 2**), which was quite different with those of *C. hupingshanesis*.

## Discussion

### *Cardamine hupingshanesis* (Brassicaceae) as a new Se-accumulator species

**Zhu et al. (2008)** [3] determined the total Se contents in five staple plant species from Yutangba, Enshi as follows: *Corn seeds*, 1.48±1.41 mg/kg DW (n = 20); *Agry wormwood*, 1.68±1.27 mg/kg DW (n = 30); *Bracken fern*, 0.63±1.61 mg/kg DW (n = 57); *Central China dryoathyrium*, 0.48±0.72 mg/kg DW (n = 39) and *Hupeh beautyberry*, 0.19±0.03 mg/kg DW (n = 5). But in the present study, *C. hupingshanesis* collected from the same study area in Enshi accumulated Se as high as 432±471 mg/kg DW (n = 27) in leaves, 425±447 mg/kg DW (n = 27) in stems and 462±709 mg/kg DW (n = 19) in roots. In fact, *C. hupingshanesis* could be identified as a new Se accumulator, or Se secondary accumulator, which could grow on soils contaminated with moderate levels of Se (10–70 mg/kg DW) in the Se mine drainage area, and accumulate Se up to 100–1000 mg/kg DW [23]–[25]. Moreover, *C. hupingshanesis* could accumulate Se up to 1965±271 mg/kg DW in leaves, 1787±167 mg/kg DW in stem and 4414±3446 mg/kg DW in roots near Se mine tailing (Sampling site YTB-3), which were comparable to those of typical Se hyperaccumulating plants, *Stanleya pinnata* (prince's plume) (1000–15000 mg Se/kg DW) and *Astragalus bisulcatus* (twogrooved milkvetch) (about 6000 mg Se/kg DW) [11]–[14], [26].

To characterize the Se-translocation from soil to plant, the bio-concentration factor (BCF) of Se was calculated (**Table 3**). *C. hupingshanesis* displayed exceptionally high BCF values of 85 in roots on site of YTB-3, 39 in stem and 38 in leaves on site of YTB-4, indicating soil Se could be efficiently taken up by it. Overall, *C. hupingshanesis* had BCFs with ranges of 9.31±9.07 (n = 28) in leaves, 8.70±7.69 (n = 28) in stems and 10.71±16.90 (n = 24) in roots. In contrast, *L. turcz* had relatively low BCFs values of <2, significantly lower (K-S test, P<0.01) than those of *C. hupingshanesis* (**Table 3**). In fact, most plant species, including forages, crops and grasses, typically accumulate less than 25 mg Se/kg DW when growing on seleniferous soil with Se concentrations of 10 mg/kg DW [23], [27], [28]. In *Brassica juncea* (Indian mustard) and *Brassica napus* (Canola) shoots, the BCFs were less than 10 [29]–[31]. However, *Stanleya pinnata* (prince's plume) and *Astragalus bisulcatus* (twogrooved milkvetch) had BCFs typically >100 [32]–[36].

**Table 3:** Values of Bioconcentration factor (BCF), Stem/Root ratios and Leaf/Root ratios of Se concentrations in plants.

Plant species	Tissue (sample numbers)	BCF	Leaf_con_/Root_con_	Stem_con_/Root_con_
*Cardamine hupingshanesis*	Leaf (n = 28)	9.31±9.07	1.76±0.98	1.72±0.95
	Stem (n = 28)	8.70±7.69		
	Root (n = 24)	10.71±16.90		
*Ligulariafischeri (Ledeb.) turcz*	Root (n = 6)	0.93±0.68	1.37±0.95	0.72±0.22
	Stem (n = 6)	0.77±0.72		
	Leaf (n = 6)	1.65±1.95		

The translocation of Se from root to shoot by plants could be indicated by Se concentrations from stem/root and leaf/root ratios; thus calculated values were summarized in **Table 3**. The stem_con_/root_con_ values and the leaf_con_/root_con_ values were very close in *C. hupingshanesis* with 1.72±0.95 (n = 24) and 1.76±0.98 (n = 24), respectively, which likely indicated that stems of *C. hupingshanesis* were not effective Se channels. Moreover, the stem_con_/root_con_ values in *C. hupingshanesis* were significant higher (Two Sample t Test, t = 2.50, Df = 20, P = 0.02) than those in *L. turcz*, although there were no significant (Two Sample t Test, T = 0.84, Df = 20, P = 0.41) differences on the leaf_con_/root_con_ values, which revealed that *C. hupingshanesis* had much better performance to transport Se from root to stem. It is known that translocation of Se from root to shoot depends on the forms of Se supplied from the soil, in which selenate is much more easier to transport, then selenite and SeMet [23], [37], [38]. Based on previous studies conducted by **Zayed et al. (1998)** [39], the bioavailable chemical species of Se in underlying sediments in the present study likely were selenate since the shoot Se/root Se ratios were greater than 1.4.

### Selenocystine accumulation in plants

Selenocysteine (SeCys) is a key component in Se metabolism for plant, which will be incorporated into proteins, transformed to elemental Se (Se^0^), or converted to selenomethionine (SeMet) or selenomethylcysteine (SeMeCys), then methylated to volatile Se compounds as dimethylselenide (DMSe) or dimethyldiselenide (DMDSe) [16], [23], [40]. However, it is uncommon that plants accumulate Se primarily in the form of SeCys because SeCys will misincorporate into proteins by replacing cysteine (Cys) to cause toxicity [27], [41], [42]. Usually, nonaccumulating plants such as *Arabidopsis* and Secondary accumulating plants such as Indian mustard (*Brassica juncea*) store Se mainly as selenate when the plants are fed with selenate. In contrast, selenate-supplied biofortified vegetables, such as garlic (*Allium sativum*), onion (*Allium cepa*), leek (*Allium ampeloprasum*) and broccoli (*Brassica oleracea*), store Se predominantly as SeMeCys [6], [16], [43]. SeMet is the predominant Se species in most grains, such as wheat, barley and rye [44]. Even in hyperaccumulating plants, such as prince's plume (*Stanleya pinnata*) (Fabaceae) and twogrooved milkvetch (*Astragalus bisulcatus*) (Brassicaceae), around 90% of the accumulated Se is present as SeMeCys in specialized cells in the leaf epidermis or in leaf hairs [13], [45]. **Freeman et al. (2010)** [15] further identified that *Stanleya albescens* (Brassicaceae), as a secondary Se accumulator, accumulated Se mainly as free amino acid selenocystathionine (SeCyst). Therefore, this is the first study reporting up to 99% of total Se as SeCys_2_ or SeCys in a higher plant.

In fact, although Se is an essential human and animal nutrient that is needed for several proteins, such as glutathione peroxidase, thioredoxin reductase and at least 23 other essential selenoproteins, no such requirement for Se has been shown for higher plants [23], [40]. Generally, the metabolism pathway of Se in non-hyperaccumulating higher plants is coupled with S metabolism pathways [23], [40]. Many studies indicated that selenocysteine methyltransferase (SMT) plays an important role in Se metabolism in Se hyperaccumulators, such as *Astragalus bisulcatus* (Fabaceae) and *Stanleya pinnata* (Brassicaceae); SMT methylates SeCys and diverts Se away from proteins, thereby reducing Se toxicity in plant, which could provide Se tolerance and hyperaccumulation [27], [32], [41]. Interestingly, SMT enzyme from *A. bisulcatus* has been successfully overexpressed in two different non-hyperaccumulating plants, *Arabidopsis thaliana* and *Brassica juncea* (Indian mustard), via transgenic approaches, confirming that SMT is one of key enzymes for Se hyperaccumulation [35], [36], [46]. A study on the molecular mechanism of Se tolerance and hyperaccumulation in *S. pinnata* showed that it related with a constitutively higher expression of genes involved in sulfur assimilation, antioxidant activities, defense, and response to (methyl)jasmonic acid, salicylic acid, or ethylene [15].

In the present study, SeMet and SeMeCys were detected in much lower proportions than SeCys_2_, which indicated SeCys methyltransferase, cysthathionine-γ-synthase, cysthathionine-β-lyase, and methionine synthase did not play a significant role in Se accumulation or tolerance for *C. hupingshanesis*. So what is the possible mechanism for *C. hupingshanesis* to survive in the Se-mine drainage area of Enshi? The accumulation Se in peripheral tissues of young leaves and reproductive organs may be a strategy to accumulate high concentrations of Se as SeCys_2_ [45], [47]–[55]. Also, rhizospheric bacteria may contribute to the high ability to accumulate Se or SeCys_2_ in tissues [56], [57]. Further study should be carried out on distinguishment special SeCys_2_ synthase or enhancement by rhizospheric microorganisms to explain high SeCys_2_ accumulation in *C. hupingshanesis*.

## Conclusions

In the Se-mine drainage area from Enshi, China, *Cardamine hupingshanesis* (Brassicaceae) was identified as a new Se secondary accumulator species. It could accumulate Se to concentrations ranging as 239±201 mg/kg DW in roots, 316±184 mg/kg DW in stems, and 380±323 mg/kg DW in leaves, with the underlying top sediment Se contents of 35.79±17.95 mg/kg DW. Particularly, this plant species could accumulate Se up to 1965±271 mg/kg DW in leaves, 1787±167 mg/kg DW in stem and 4414±3446 mg/kg DW in roots, which were comparable to typical Se hyperaccumulating plants, *Stanleya pinnata* and *Astragalus bisulcatus*. Furthermore, *C. hupingshanesis* had a high ability to accumulate Se from sediment with bio-concentration factors of about 10, and it could accumulate up to 99% of total Se as SeCys_2_ in plant tissues without showing phytotoxic symptoms. The current Se metabolisms knowledge could not give a reasonable explanation on it. More research should be needed to elucidate the possible mechanism on it.
